# Health and Education in Yugo-Slavia

**Published:** 1921-09-24

**Authors:** 


### Health and Education in Yugo-Slavia.

This newest among the kingdoms of the world has inaugurated its government by the appointment of a Health Minister. Dr. Andrea Stampar, Chief of the Division of Social Hygiene in Yugo-Slavia, finds himself confronted with an ignorance on all health matters against which it is extremely difficult to contend, owing to the fact that few of the people can read, while there is a great scarcity both of teachers and schools. The principal mode of reaching the intelligence of the people is through pictures on the screen. A hundred cinematograph films are in the possession of the Ministry, and health dramas have been organised. Very promising work is being done in the way of controlling epidemics. To this end a special school, with a six-months' course, has been opened for training sanitary officers. As soon as they are trained they are sent out with the necessary apparatus to form disinfecting units, and there are three disinfecting trains ready to leave for any part of the country in which rapid disinfection is called for. There are ten bacteriological stations devoted to the study of infectious diseases, and an institute is being created for the supply of serums; dispensaries are being founded for combating tuberculosis; and fifteen health visitors who are to serve in them are in course of training. Malaria is a serious problem, infecting, it is estimated, some million persons, but a campaign has been inaugurated against it with the help of the American commission. Drunkenness takes a heavy toll on the inhabitants and temperance propaganda has been instituted in the schools, while a law has been passed forbidding the sale of spirits from 6 p.m. Saturday to midday on Monday, and during elections. We are indebted to the *International Public Health Journal* for these particulars which throw light on the energy and public spirit which Yugo-Slavia is devoting to the improvement of health conditions among its citizens.

